# Probabilistic Protein Function Prediction from Heterogeneous Genome-Wide Data

**DOI:** 10.1371/journal.pone.0000337

**Published:** 2007-03-28

**Authors:** Naoki Nariai, Eric D. Kolaczyk, Simon Kasif

**Affiliations:** 1 Bioinformatics Program, Boston University, Boston, Massachusetts, United States of America; 2 Department of Mathematics and Statistics, Boston University, Boston, Massachusetts, United States of America; 3 Department of Biomedical Engineering, Boston University, Boston, Massachusetts, United States of America; University of Washington, United States of America

## Abstract

Dramatic improvements in high throughput sequencing technologies have led to a staggering growth in the number of predicted genes. However, a large fraction of these newly discovered genes do not have a functional assignment. Fortunately, a variety of novel high-throughput genome-wide functional screening technologies provide important clues that shed light on gene function. The integration of heterogeneous data to predict protein function has been shown to improve the accuracy of automated gene annotation systems. In this paper, we propose and evaluate a probabilistic approach for protein function prediction that integrates protein-protein interaction (PPI) data, gene expression data, protein motif information, mutant phenotype data, and protein localization data. First, functional linkage graphs are constructed from PPI data and gene expression data, in which an edge between nodes (proteins) represents evidence for functional similarity. The assumption here is that graph neighbors are more likely to share protein function, compared to proteins that are not neighbors. The functional linkage graph model is then used in concert with protein domain, mutant phenotype and protein localization data to produce a functional prediction. Our method is applied to the functional prediction of *Saccharomyces cerevisiae* genes, using Gene Ontology (GO) terms as the basis of our annotation. In a cross validation study we show that the integrated model increases recall by 18%, compared to using PPI data alone at the 50% precision. We also show that the integrated predictor is significantly better than each individual predictor. However, the observed improvement vs. PPI depends on both the new source of data and the functional category to be predicted. Surprisingly, in some contexts integration hurts overall prediction accuracy. Lastly, we provide a comprehensive assignment of putative GO terms to 463 proteins that currently have no assigned function.

## Introduction

Functional annotation of genes is a fundamental problem in computational and experimental biology. The problem can be solved at various levels of resolution ranging from identifying high level processes where a given protein might be associated with, to discovery of the cell specific protein-ligand interaction targets of a protein in different biological conditions. The most established and reliable methods for protein function prediction are based on sequence similarity using BLAST [1] and profile methods such as PFAM [2], and PSI-BLAST [1]. Other still evolving methods that are too numerous to list include gene fusion information [3], and phylogenetic profiling [4], [5]. Emergent methods that elucidate function from a variety of high-throughput experimental screens have become particularly attractive recently due to the reduced cost of conducting genome-wide functional screens. Genomic and proteomic data sets, including gene expression and protein-protein interaction (PPI) data, are becoming increasingly available for a growing array of organisms. Driven by the hypothesis that co-expressed genes might participate in related biological processes, clustering gene expression profiles across diverse conditions can be used to assign protein function [6]–[8]. Using PPI data to assign protein function has been extensively studied. These algorithms are often based on the “guilt by association” principle that suggests that interacting neighbors in protein-protein interaction (PPI) networks might also share a function [9]–[11]. Since such genome-wide data sets are inherently noisy, and each type of data captures only one aspect of cellular activity (e.g. gene expression data measure mRNA levels of transcriptionally induced genes, and PPI data suggest a feasible physical interaction between proteins), it is appealing to combine such heterogeneous data in an effort to improve the coverage and accuracy of protein function prediction.

Bayesian network methodologies for data integration have been explored [12]–[14] in a number of systems for predicting protein-protein interactions and protein function similarity. These approaches calculate the posterior probability that each pair of genes *i* and *j*, has a functional relationship, given the various types of genome-wide data. These algorithms output a functional linkage graph [3], [15] in which an edge between two nodes (genes) represents functional similarity with a reliability score (probability) assigned to each edge. However, using these probabilistic networks to produce a functional assignment remains a hard computational problem. For instance, one approach for protein function annotation based on Markov random fields (MRFs) has been previously investigated [10]. An integrated MRF approach that includes network structures (PPI network and co-expression network) and protein domain information to predict protein function has also been proposed [16]. There, the authors used Gibbs sampling to estimate the probability that a protein has a particular function. Machine learning methods based on support vector machines have been investigated in several projects [17], [18]. In fact, it is rather obvious that if we treat the prediction of function based on each modality as an expert, then any of the popular classification methods (decision trees, boosting, and weighted majority) can in principle be used for “integration” of these predictions. However, given the currently sparse data using complex representations for prediction might lead to overfitting.

In this paper, our contribution is twofold. First, we propose a simple and relatively transparent probabilistic model for protein function prediction that allows us to efficiently calculate the posterior probability that each gene has a particular function, given various types of genome-wide data. Second, we analyze the effect of combining the heterogeneous data sources in a substantially more comprehensive manner than has been done to date, with the goal of better understanding just which types of genes benefit most from the integration of which types of data sources. In particular, we develop a relatively simple yet useful method to integrate functional linkage graphs with categorical information. The functional linkage graphs are constructed from PPI data and gene expression data. As usual the assumption here is that physically interacting proteins or co-expressed genes are more likely to share protein functions than a randomly selected pair of proteins [10]. Categorical features for each protein, including protein motifs, knockout phenotype, and localization information are captured based on predictive sources of evidence available from the MIPS database [19]. Using Bayesian networks framework, this categorical information is then combined with functional linkage graphs constructed from PPI data and gene expression data to generate functional predictions. Our method is applied to the functional prediction of proteins in yeast (*Saccharomyces cerevisiae*). Our methodology combines PPI data, gene expression data, protein motif information, mutant phenotype data, and protein localization data, while using Gene Ontology (GO) “biological processes” terms [20] as the basis for functional annotation. The long term goal of this research is to develop a probabilistic language to specify which proteins might be active in a given biological process based on the type of interacting partners they have, protein motifs, or transcriptional profiles.

By combining five types of data, the number of correctly recovered known gene-term associations is increased by 18% at the same precision (50%), compared to using PPI data alone. We specifically focused on certain points on the ROC curve in our analysis that we believe are potentially feasible for follow-ups on the prediction in experimental labs. We show that by adding different types of genome-wide data, different types of the GO terms that are specific for the type of information are newly recovered. Also, by conducting robustness analysis of the integration model to PPI edge removal, we provide a novel perspective on the amount of PPI data necessary to obtain high prediction accuracy by the integration model. In that analysis, we find some conditions where integration actually hurts performance rather than improving accuracy. Plausible functions are assigned to 463 currently unannotated proteins by our method, and we discuss some of these novel assignments.

## Methods

### 2.1 Data preparation

#### 2.1.1 Protein-protein interaction data

From the GRID database [21], 31201 non-redundant protein-protein interactions among 5151 yeast *Saccharomyces cerevisiae* genes are extracted. We eliminated self-self interactions and duplicated protein interaction pairs from the database to construct a PPI functional linkage graph [3], [15], in which an edge between two nodes (proteins) represents evidence for protein function similarity.

#### 2.1.2 Gene expression data

Four types of gene expression data, the Rosetta compendium data [22], cell cycle data [23], stress-response data [24], and DNA-damage data [25] are used in this paper. For each type of gene expression data, Pearson correlation coefficients for all combinations of genes are calculated, and gene pairs whose correlation coefficient is larger than 0.85 are selected as “co-expressed pairs” for each type of gene expression data. We obtained 1783, 645, 10654 and 31827 gene pairs from the Rosetta data, cell cycle data, stress-response data and DNA damage data, respectively. The false discovery rate (FDR) [26], [27] for each threshold is less than 10^−10^, indicating that for each experiment we are only using a set of declared co-expressed pairs for which a false declaration is exceedingly unlikely. Finally, 38151 non-redundant gene pairs are obtained from the combined gene pairs to construct a co-expression functional linkage graph.

#### 2.1.3 Protein motif information

From the MIPS [19] database, 2678 protein-motif associations (e.g. YCR065W protein has “Fork head domain signatures and profile” motif) are extracted, covering 2179 proteins across 992 motif categories. If a protein has a specific protein motif, this can increase the probability that the protein has a specific protein function. We describe how to integrate this category information for protein function prediction in Section 2.3.

#### 2.1.4 Gene knock-out phenotype data

From the MIPS [19] database, 3013 protein-phenotype associations (e.g. YPR185W deletion mutant exhibits “Starvation sensitivity”) are obtained, covering 1460 proteins across 175 mutant phenotype categories.

#### 2.1.5 Protein localization data

From the MIPS [19] database, 5191 protein-localization data (e.g. YPR191W protein localizes at “Mitochondrial inner membrane”) are obtained, covering 4076 proteins across 41 cellular location categories.

#### 2.1.6 GO term data

From the 06/03/2006 version of the Yeast SGD database [28], 107636 gene-term GO assignments are obtained, in which there are 6289 genes and 1965 ‘biological process’ terms in total. For each gene-term association, we expanded the label in the GO hierarchy to include all ‘is-a’ and ‘part-of’ ancestors of each GO term. Labels that appear more than 300 times among the 6289 genes are excluded for further analysis, on the assumption that such terms are too broad for protein function prediction. Also labels that appear less than five times among the genes are excluded, since they do not constitute a sufficiently large enough sample to make reliable predictions.

From PPI data and gene expression data, two different functional linkage graphs are obtained. Here, an edge in each functional linkage graph shows that the two nodes (proteins) are a member of the constructed pairs in each data set. For each GO label *t* and for each functional linkage graph *l*, we calculate *p*_1_^(*l*)^, the probability that a protein has label *t*, given that the interacting partner has label *t*. This *p*_1_^(*l*)^ is expected to be higher than *p*_0_^(*l*)^, the probability that the protein has label *t*, given that the interacting partner does not have label *t*. Here, a *X* ^2^ test was performed to ensure that *p*_1_^(*l*)^ and *p*_0_^(*l*)^ were statistically different using a Bonferroni-corrected *p*-value of 0.001*/T* , where *T* is the number of terms tested in each data set.

### 2.2 Categorical features of proteins

Proteins can be associated with categorical features according to different types of categorical information. The categorical features that are used in our predictive methodology are defined below.

- Protein motif (domain): Random variable *d_i_* is associated with a protein where *d_i_* = 1 if the protein contains domain *d_i_*, and *d_i_* = 0 otherwise. A feature vector **d** = (*d*_1_, *d*_2_,…, *d_qd_*)^T^ is defined for each protein, where *q_d_* is the total number of protein motif features (*q_d_* = 992 in our case).
- Phenotype: Random variable *p_i_* is associated with a protein where *p_i_* = 1 if the gene knockout exhibits phenotype *p_i_*, and *p_i_* = 0 otherwise. A feature vector **p** = (*p_1_*, *p_2_*,…, *p_qp_*)^T^ is defined for each protein, where *q_p_* is the total number of phenotype features (*q_p_* = 175 in our case).
- Protein localization: Random variable *l_i_* is associated with a protein where *l_i_* = 1 if the protein localizes in *l_i_*, and *l_i_* = 0 otherwise. A feature vector **l** = (*l*_1_, *l*_2_,…, *l_ql_*)^T^ is defined for each protein, where *q_l_* is the total number of localization features (*q_l_* = 41 in our case).

Naturally, a protein can have several features at the same time. Our aim is to integrate these sources of evidence in a smooth fashion to improve the accuracy and coverage of the functional predictors based on the assumption that if a protein has specific features, then this can increase the probability to infer specific protein functions.

### 2.3 Computing the posterior probability of function using graphs and features

For each protein *i* and GO term *t*, a Boolean random variable *L_i,t_* is associated, where *L_i,t_* = 1 if *i* is labeled with *t*, and *L_i,t_* = 0 otherwise. We want to calculate the probability of *L_i,t_* = 1 for all combinations of *i* and *t*, given the structure of functional linkage graphs constructed above, and the category features that the protein *i* has, and all the assignments of GO terms to the other proteins. We assume that probability distribution for the labeling *L_i,t_* = 1 is conditionally independent of all other nodes given the functional annotation of the neighbors and category information of the protein.

We want to calculate the posterior probability given functional linkage graphs and category features of a protein *P*(*L_i,t_* = 1|*N_i_* ^(l)^,…, *N_i_* ^(*m*)^, *k_i,t_* ^(l)^,…, *k_i,t_* ^(*m*)^, **c** *_i_* ^(l)^,…, **c** *_i_* ^(*n*)^), where *N_i_* ^(l)^(*l* = 1,…, *m*) is the number of graph neighbors (excluding unannotated neighbors) of gene *i* in a functional linkage graph *l*, *k_i_* ^(l)^(*l* = 1,…, *m*) is the number of the neighbors of gene *i* which are labeled with term *t* in the graph *l*, *m* is the number of different types of functional linkage graphs, **c** *_i_* ^(*j*)^(*j* = 1,…, *n*) is the feature vector that the gene *i* has for a category feature type *j*, and *n* is the number of different types of categories (not the number of features). For example, in this paper, *N_i_* ^(1)^ and *k_i_* ^(1)^ is the number of neighbors of gene *i*, and the neighbors that have term *t* in a PPI network, respectively. *N_i_* ^(2)^ and *k_i_* ^(2)^ is the number of the neighbors and the neighbors that have term *t* in a co-expression network, respectively. **c** *_i_* ^(1)^, **c** *_i_* ^(2)^ and **c** *_i_* ^(3)^ are feature vectors that gene *i* has for the three types of categories, i.e., protein motif feature vector **d**, mutant phenotype feature vector **p**, and localization feature vector **l**, respectively (Section 2.2).

Applying Bayes' theorem, the posterior probability that gene *i* has function *t P*(*L_i,t_* = 1|*N_i_* ^(1)^,…, *N_i_* ^(*m*)^, *k_i,t_* ^(1)^,…, *k_i,t_* ^(*m*)^, **c** *_i_* ^(1)^,…, **c** *_i_* ^(*n*)^) can be rewritten (with omitting subscript *i* and *t*) as:
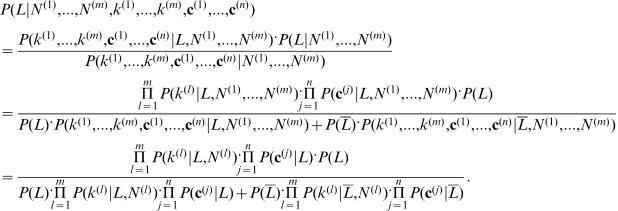
1


Here, we assume that the probability distribution of *L_i,t_* is independent of the number of graph neighbors *N* ^(1)^,…, *N* ^(*m*)^, hence *P*(*L*|*N* ^(1)^, …, *N* ^(*m*)^) =  *P*(*L*). Also, we assume that *k* ^(1)^,…, *k* ^(*m*)^, **c** ^(1)^,…, **c** ^(*n*)^ are conditionally independent of each other, given *L* and *N* ^(1)^,…, *N* ^(*m*)^. This assumption is similar to the case in a Naive Bayes classifier. It is natural to assume that *k* ^(*l*)^ is conditionally independent of *N* ^(1)^,…, *N* ^(*m*)^ except *N* ^(*l*)^, given *L*. Also, **c** ^(*j*)^ is conditionally independent of *N* ^(1)^,…, *N* ^(*m*)^, given *L*. Now we need to calculate each decomposed product in (1).

*P*(*k* ^(*l*)^|*L,N* ^(l)^) is the probability that *k* ^(*l*)^ neighbors are labeled with *t* out of *N* ^(*l*)^ neighbors in a graph *l*, given that the gene *i* is labeled with *t*. Here we assume a binomial distribution [10] and calculate this probability as 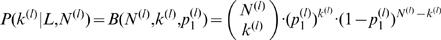, where *p*_1_^(*l*)^ is the probability that a protein *i* has label *t*, given that an interacting partner has label *t* within a functional linkage graph *l*, which is pre-calculated by training data (Section 2.1.6). Similarly, 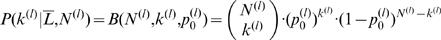, where *p*_0_^(*l*)^ is the probability that a protein *i* has label *t*, given that an interacting partner does not have label *t* within a functional linkage graph *l*.

*P*(c^(*j*)^|*L*) is the probability that a gene *i* has feature vector c^(*j*)^ given that the gene *i* has term *t*. *P*(*L*) is the prior probability that the gene *i* has term *t*. This is calculated as *P*(*L*) = *f*, where *f* is the frequency of term *t* among genes.

Hence the neighborhood function (1) becomes:
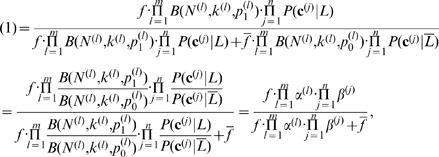
2where 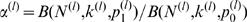 and 
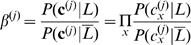
, assuming conditional independence between the feature vectors. Here, *P*(*c_x_* ^(*j*)^  = 1|*L*) = (# of *t*-labeled genes that have a feature *c_x_* ^(*j*)^)/(# of *t*-labeled genes), and *P*(*c_x_* ^(*j*)^ = 1|*L* ¯) = (# of genes that are not labeled with *t* and have a feature *c_x_* ^(*j*)^)/(# of genes that are not labeled with *t*). Since we do not want lack of information to affect protein function prediction, we assume 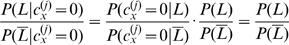 hence *P*(*c_x_* ^(*j*)^  = 0| *L*)/*P*(*c_x_* ^(*j*)^ = 0|*L* ¯) = 1. For example, suppose that the current genome-wide data do not contain information that a protein is localized in mitochondria. However, this does not necessary mean that the protein does not localize in mitochondria. The reason might be that just lack of the currently available data.

## Results

The integration algorithm described in the Methods section is evaluated on the task of predicting protein functions for *Saccharomyces cerevisiae*. The algorithm fuses probabilities obtained from diverse data sources including PPI, gene expression, protein motif information, gene knock-out phenotype, and protein localization. The functional annotations used to train our models are based on the GO category and are obtained from Yeast SGD database [28]. The algorithm is first validated on known protein-term associations by a 5-fold cross validation methodology. We also conduct robustness analysis to understand the effect of removal of PPI edges on the accuracy of the prediction with one or more data sources. Finally, we predict protein function of unannotated genes.

### 3.1 Cross Validation Analysis of Prediction Accuracy

**Figure 1:**
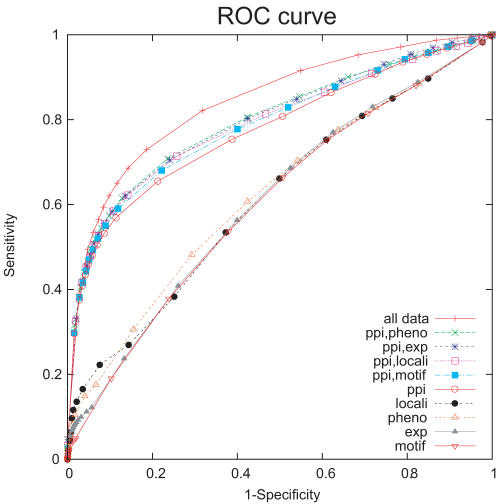
The ROC curve of recall experiment by 5-fold cross validation. Sensitivity is defined as #TP/(#TP+#FN), and specificity is defined as #TN/(#FP+#TN).

First, we attempted to predict known protein-term associations by 5-fold cross validation. For each gene *g* and term *t*, the probability that gene *g* has term *t* is calculated based on equation (2), given that we know every other gene-term associations in the training set. It is predicted that gene *g* has term *t* if the probability exceeds a specified threshold. A positive *g-t* association set is obtained from the GO “biological processes” data, and negative *g-t* association set is defined as follows: If the association is not in the positive set, and *g* is annotated with at least one biological process *t*, and *t* is neither ancestor nor descendant of the known function in the GO hierarchy. Figure 1 presents a ROC curve of function prediction by different combinations of each data sources. Sensitivity is defined as #TP/(#TP+#FN), which corresponds to recall, and specificity is defined as #TN/(#FP+#TN), which corresponds to precision. For varying posterior probability cut-off, 1-specificity and sensitivity is plotted. The result shows that by combining the five specific types of data described above, protein functions can be predicted more accurately, compared to each data source alone.

**Figure 2:**
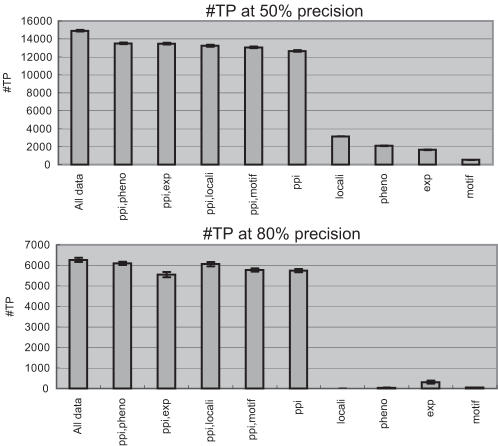
#TP at 50% precision (Upper) and #TP at 80% precision (Lower). Here, *ppi*, *exp*, *motif*, *pheno*, and *locali* corresponds to PPI, gene expression, protein motif, phenotype, and localization data, respectively.

Figure 2 summarizes the impact that data integration has on protein function prediction sensitivity at a fixed precision (50% and 80%). The error bar shows the standard deviation of 10 independent cross-validation experiments. At the 50% precision, 14906 known protein-term associations can be recovered on average by combining five types of data. On the other hand, when we use PPI data alone, 12662 associations can be recovered on average. Our integrated method thus realizes an 18% increase in the number of functional predictions for genes at the 50% precision. At the 80% precision, the combination of all data (PPI, gene expression, protein motif, mutant phenotype, and protein localization) works better than any other combinations and other single source of data. However, at the 80% precision, combining PPI data with one other data source shows a little improvement in predictive accuracy, suggesting that PPI data is particularly informative.

These two levels of precision, i.e., 50% and 80%, were chosen as being reasonably representative of the range of possible improvements observed in our study. In addition to the performance characteristics just described, we also examined the issue of falsely predicted proteins, as a function of the threshold applied to posterior probabilities. Using the method of [29], the rate of false discoveries, for the classifier integrating all data sources, was estimated to be 0.13 and 8.4×10^−6^, respectively, at the 50% and 80% precision levels.

Next, we analyzed whether prediction accuracy depends upon the functional category to be predicted. It is expected that the prediction performance of specific GO terms depends on what kinds of data sources one uses. Table S1 (in Supporting Information) shows the list of GO terms, which are improved by adding gene expression data in addition to PPI data at the 50% precision for each GO term prediction. Here GO terms are listed, of which the number of #TP is increased by at least 10, compared to that of using PPI data alone. We can see from the result that many of the improved terms are metabolism related (e.g. “amino acid and derivative metabolism”, “nitrogen compound biosynthesis”, and etc.). Since metabolic reactions are often interactions between enzymes and compounds, and such proteins (enzymes) do not necessarily have protein-protein interactions between enzymes in a same pathway, it might be difficult to reconstruct and hence predict such metabolic pathways by using PPI data alone. In this sense, measuring gene expression of enzymes and identifying co-expressed genes will be supplementary information for capturing functionally related genes. Here, the result suggests that the gene expression data actually helps to identify such metabolic components that are working in a same pathway. Table S2 shows the list of GO terms, which are improved by adding protein motif information in addition to PPI data. Here, it is interesting to see that GO terms “phosphorylation” and “phosphate metabolism” are most strikingly improved. Also, among newly recovered genes that have the GO term “transcription from RNA polymerase II promoter” by adding motif information, nine proteins have a protein motif “Zinc finger, C2H2 type, domain”. Since protein kinases and transcription factors often have specific binding domains, protein motif information are particularly useful for predicting these terms. Table S3 shows the list of GO terms, which are improved by adding phenotype data in addition to PPI data. Among the improved GO terms, “cell wall organization and biogenesis”, “cell budding”, “reproduction”, and “external encapsulating structure organization and biogenesis” might be related to phenotypes of a cell, and only listed in this table. Table S4 shows the list of GO terms, which are improved by adding localization data in addition to PPI data. Among the improved GO terms, “ion transport”, “Golgi vesicle transport”, and “vesicle-mediated transport” are cellular location specific GO terms, and these terms are only listed in this table. We can conclude from these results that by adding different types of genome-wide data, different types of GO terms that are specific for the data type can newly be predicted.

Here is an example how the combination of different types of data helps to predict protein function more specifically. Genes YKR055W, YIL118W and YJL128C have a GO term “intracellular signaling cascade”, but neither the PPI data nor the protein motif information alone can predict the GO term for the proteins. When PPI data alone is used, a GO term “signal transduction”, which is a parent of “intracellular signaling cascade” in the GO hierarchy and hence a broader term, can be predicted. However, when both PPI data and protein motif information are used, the GO term can be predicted correctly. In this case, information that the proteins have a protein motif “protein kinases signatures and profile” or “prenyl group binding site” helps to predict more specific term “intracellular signaling cascade” correctly.

### 3.2 Robustness analysis of the integration model

**Figure 3:**
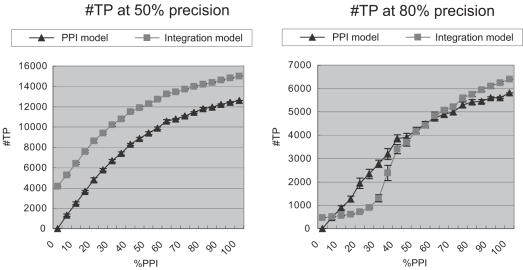
#TP at 50% precision (Left) and #TP at 80% precision (Right) with varying amount of PPI edges. At 50% precision, the integration model always wins over the PPI model. However, at 80% precision, the integration model wins only when more than 50% of original PPI edges are present.

In the recall experiment in Section 3.1, we showed that PPI data is the strongest source of evidence for protein function prediction in our model, compared to other data sources. Here, we want to know whether our integration model works well or not when the amount of PPI data is limited. In this experiment, a certain fraction of the PPI edges are randomly removed from the original PPI network, and then protein function is predicted using our integration model. Figure 3 shows the result of prediction at 50% precision (left) and 80% precision (right). Here, *x*-axis shows how much of PPI edges are present, compared to the original PPI network. For example, at *x* = 50, half of PPI edges are randomly removed from the original PPI network. The error bar shows the standard deviation of 10 independent experiments. At 50% precision, the integration model always wins, regardless of the number of PPI edges present. However, interestingly, at 80% precision, the integration model wins only when more than 50% of PPI edges are present. In other words, in order to obtain high prediction accuracy (80% precision) by the integration model, certain amount of PPI data is necessary (more than 50% of original PPI edges in this case). This result suggests that the combination of gene expression, protein motif, mutant phenotype and protein localization data is still a weak indicator of protein function, and hence need to have certain amount of PPI information (strong indicator of protein function) in order to obtain high prediction accuracy (80% precision).

### 3.3 Prediction of function unknown proteins

By integrating five types of data, we assign plausible GO terms to 463 proteins among 1481 currently unannotated yeast *Saccharomyces cerevisiae* proteins (complete list available in Supporting Information, Table S5). The threshold probability for function annotation is 0.470, in which we expect 50% precision for the protein function prediction from the cross validation experiment in Section 3.1.

Among the predicted function of unannotated proteins, recent literature reported [30] that YBL028C, YBR271W, YCR016W, YJR003C, YDL167C, YDR361C, YIL096C, YIL127C, YLR449W, YMR310C, YNL022C, YNL132W, YNL175C, YGR187C, YGR283C and YOR021C as rRNA and ribosome biosynthesis (RRB) regulon. It has also been reported [31] that YLR051C encodes a protein involved in pre-rRNA processing, confirming our prediction of “ribosome biogenesis” (or related terms). Other than ribosomal proteins, recent literature reported that YAL053W participates in a cell wall biosynthesis process [32]. Since our prediction for YAL053W is “cell wall organization and biogenesis”, we can say that there is an experimental validation for the prediction. Also, it is reported [33] that YBR280C encodes a protein, which targets Aah1p for proteasome-dependent degradation. Here, our prediction for the protein “SCF-dependent proteasomal ubiquitin-dependent protein catabolism” is quite consistent with the literature.

It is confirmed here that 20 out of 463 function predictions for unannotated proteins are quite consistent with the conclusion from the recent publications. We expect that many of our predictions will turn out to be true after validation experiments.

All the biological data and a Perl program used in this analysis are available at: http://genomics10.bu.edu/nariai/yeast_func/.

## Discussion

In this paper, we propose a probabilistic method to predict protein function from multiple types of genome-wide data. Pair-wise information between proteins, such as PPI data or co-expression information is converted into a functional linkage graph, in which an edge between nodes represents evidence for protein function similarity. Category information, such as protein motif information, mutant phenotype data, and protein localization data is combined with the functional linkage graphs using a unified probabilistic framework. We showed in our 5-fold cross validation experiment that our method successfully improved prediction accuracy and coverage by integrating five types of genome-wide data. Also, by conducting robustness analysis of the integration model to PPI edge removal, we showed that there is a certain amount of PPI data necessary to obtain high prediction accuracy by the integration model. We proposed functional predictions for 463 currently unannotated proteins. One subjective aspect of our method is in the choice 0.85 in thresholding the correlation coefficients in constructing our co-expression functional linkage graph. However, we have found our results to be quite robust to this choice; for example, even much higher thresholds yield qualitatively quite similar results. In principle, a more objective choice of threshold could be made through the use of cross-validation, but this would come at the cost of an increased computational burden. Other limitations are that we assume probabilistic conditional independence between different types of functional linkage graphs and each informational category. Of course, this assumption might not always be correct in a biological sense. For example, some of physically interacting protein pairs are also co-expressed. However, previous literature has reported that Naive Bayes frequently tends to work well, and frequently better than more sophisticated classifiers, when the data are sparse compared to the dimensionality of the problem, even when the features (e.g., in our case, the functional linkage graphs and category feature vectors) are not truly conditionally independent [34], [35]. Hence, we anticipate that our method may in fact prove to be a fairly strong contender in competition, for the types of data we use, with more sophisticated methods that may follow. In addition, we assume independence between non-ancestral GO terms. Since the GO terms comprise a hierarchical structure, and there would be dependencies among the GO terms, one might want to take the dependency between the nodes into account. Also in our method, within a functional linkage graph, non-neighbors (two nodes whose distance are more than one) are not considered for functional similarity. Application of a Markov Random Field model in conjunction with belief propagation and/or sampling may address these limitations and is the subject of on-going investigation.

Although the result presented here is a case study for yeast *S. cerevisiae*, we believe that similar advances are possible for fly, worm, mouse and human where analogous resources are being compiled.

## Supporting Information

Table S1 Improved GO terms by adding gene expression data at 50% precision.
(0.02 MB XLS)

Table S2 Improved GO terms by adding protein motif data at 50% precision.
(0.02 MB XLS)

Table S3 Improved GO terms by adding phenotype data at 50% precision.
(0.02 MB XLS)

Table S4 Improved GO terms by adding localization data at 50% precision.
(0.02 MB XLS)

Table S5 Prediction result of unannotated genes. N1 is the number of neighbors in PPI network, k1 is the number of t-labeled (t is the predicted GO term) neighbors in PPI network, N2 is the number of neighbors in co-expression network, and k2 is the number of t-labeled neighbors in co-expression network.
(0.23 MB XLS)
